# The *‘Liaisons dangereuses’* Between Lung Cancer and Interstitial Lung Diseases: A Focus on Acute Exacerbation

**DOI:** 10.3390/jcm13237085

**Published:** 2024-11-23

**Authors:** Umberto Zanini, Paola Faverio, Valentina Bonfanti, Maria Falzone, Diego Cortinovis, Stefano Arcangeli, Francesco Petrella, Giovanni Ferrara, Marco Mura, Fabrizio Luppi

**Affiliations:** 1 Department of Medicine and Surgery, University of Milano-Bicocca, SC Pneumologia, Fondazione IRCCS “San Gerardo dei Tintori”, 20900 Monza, Italy; 2 Department of Medicine and Surgery, University of Milano-Bicocca, SC Oncologia, Fondazione IRCCS “San Gerardo dei Tintori”, 20900 Monza, Italy; diego.cortinovis@unimib.it; 3 Department of Medicine and Surgery, University of Milano-Bicocca, SC Radioterapia, Fondazione IRCCS “San Gerardo dei Tintori”, 20900 Monza, Italy; 4 Department of Medicine and Surgery, University of Milano-Bicocca, SC Chirurgia Toracica, Fondazione IRCCS “San Gerardo dei Tintori”, 20900 Monza, Italy; 5 Division of Pulmonary Medicine, University of Alberta, and Alberta Health Services, Edmonton, AB T6G 2B7, Canada; 6 Division of Respirology, Western University, London, ON N6A 3K7, Canada

**Keywords:** interstitial lung disease, lung cancer, acute exacerbation, prognosis

## Abstract

Patients with interstitial lung disease (ILD) are about five times more likely to develop lung cancer than those without ILD. The presence of ILD in lung cancer patients complicates diagnosis and management, resulting in lower survival rates. Diagnostic and treatment procedures needed for cancer can increase the risk of acute exacerbation (AE), one of the most severe complications for these patients. Bronchoscopic techniques are generally considered safe, but they can trigger AE-ILD, particularly after cryoprobe biopsies. Surgical procedures for lung cancer, including lung biopsies and resections, carry an elevated risk of AE-ILD. Postoperative complications and mortality rates highlight the importance of meticulous surgical planning and postoperative care. Furthermore, cancer treatments, such as chemotherapy, are all burdened by a risk of AE-ILD occurrence. Radiotherapy is important for managing both early-stage and advanced lung cancer, but it also poses risks. Stereotactic body radiation and particle beam therapies have varying degrees of safety, with the latter potentially offering a lower risk of AE. Percutaneous ablation techniques can help patients who are not eligible for surgery. However, these procedures may complicate ILD, and their associated risks still need to be fully understood, necessitating further research for improved safety. Overall, while advancements in lung cancer treatment have improved outcomes for many patients, the complexity of managing patients with concomitant ILD needs careful consideration and multidisciplinary assessment. This review provides a detailed evaluation of these risks, emphasizing the need for personalized treatment approaches and monitoring to improve patient outcomes in this challenging population.

## 1. Introduction

Interstitial lung disease (ILD) refers to a group of over 200 conditions marked by inflammation and scarring in the lungs, with the most representative diseases being sarcoidosis and idiopathic pulmonary fibrosis (IPF) [1]. IPF is a progressive form of interstitial lung disease marked by fibrotic tissue accumulation in the lungs, impairing lung function [1]. The disease progression varies, and its prognosis is generally poor, with a median survival of 3 to 5 years after diagnosis [2]. Moreover, patients with ILD are often older and have other health conditions that can affect their survival [3,4].

Lung cancer, including both small-cell lung cancer (SCLC) and non-small-cell lung cancer (NSCLC), is the leading cause of mortality worldwide [5,6]. ILD patients have a five-fold higher risk of developing lung cancer compared to the general population since they have common risk factors, particularly cigarette smoking and fibrosis parenchymal changes [7,8,9]. Additionally, lung cancer is a predictor of mortality, with an estimated median survival of 96.0 months in patients without lung cancer and 26 months in patients with [10]. Managing lung cancer in patients with ILD is particularly challenging due to the heightened risk of severe complications, including the increased likelihood of the occurrence of an ILD acute exacerbation (AE), characterized by new widespread alveolar abnormalities and high mortality ranging from 50% to 80% [8]. Various factors, including the progression of functional, radiological, and clinical indicators, together with the severity of ILD at the time of diagnosis, can significantly increase the risk of AE in ILD patients affected by lung cancer [11].

The current literature on the impact of AE in patients with ILD and lung cancer is mainly based on retrospective and anecdotal data. Additionally, differentiating AE-ILD from other complications during treatment, such as drug-induced lung toxicity or radiation pneumonia, can be challenging for patients with ILD and lung cancer [12,13]. Our review aims to provide a synthesis of the current literature on AE in ILD patients with lung cancer, investigating its prevalence and clinical implications.

## 2. Defining Acute Exacerbation of Interstitial Lung Disease

In 2007, Collard and colleagues, in a consensus statement aimed to provide a standard definition of AE-IPF, defined this event as an acute, clinically significant deterioration of unidentifiable cause in a patient with underlying IPF, and distinct from acute respiratory worsening related to a known cause [14]. Although this statement was the first to address AE-IPF diagnosis, the proposed definition was difficult to apply in clinical practice, mainly because of the difficulty of excluding all potential known causes of acute respiratory failure in severely ill patients. A main controversial diagnostic issue was related to the role of bronchoalveolar lavage (BAL) in identifying infectious agents with a risk of worsening hypoxemia with bronchoscopy in non-intubated patients with severe respiratory failure [15].

The difficulty in defining an event as AE-IPF based on these criteria, together with the concept that ‘idiopathic’ AE-IPF may be indistinguishable both clinically and prognostically from that induced by known causes of acute lung injury, including pulmonary infection, led an expert working group to revise the previous document, published in 2016, with the aim to update the diagnostic criteria for AE-IPF and facilitate their application in clinical practice. The major differences with the previous statement include the exclusion of the term ‘idiopathic’ from the definition of AE-IPF, the change of the range for AE diagnosis from 30 days to ‘typically less than 1 month’, and the exclusion of acute respiratory failure caused by cardiac failure or fluid overload, because of its better prognosis, together with other extrapulmonary causes, such as pneumothorax, pleural effusion, and pulmonary embolism. 

However, AE is not restricted to IPF but may also complicate the course of other ILDs, including AE of rheumatic diseases (RDs) [16,17]. Unfortunately, there is not yet a specific definition of AE-ILD in non-IPF ILDs. In most studies, the diagnostic criteria applied to define an AE in non-IPF patients refer to the criteria indicated for IPF [18].

## 3. Differential Diagnoses of AE-ILD

While AE-ILD is a severe complication, other diseases in the differential diagnosis can make recognition difficult, including drug-induced lung toxicity, radiation pneumonitis, and infections, as shown in Table 1.

Drug-induced (DI) lung toxicity is an increasing cause of ILD [13]. A Fleischner Society paper outlines three key diagnostic criteria for DI-ILD: new pulmonary opacities on CT, temporal association with systemic therapeutic agent use, and exclusion of other causes of ILD [19]. The drugs that more frequently can cause lung toxicity include disease-modifying antirheumatic drugs (DMARDs), antiarrhythmics, antimicrobial, and antineoplastic drugs, particularly immune checkpoint inhibitors (ICIs) and, more recently, the antibody-drug conjugates (ADCs) [20,21,22]. 

Radiation-induced lung injury (RILI) is a complication of radiotherapy and is related to treatment factors, such as radiation dose, fractionation, dose rate, and lung volume; this condition could be complicated or exacerbated by the concomitant or sequential use of antiblastic agents (i.e., pneumonitis recall signs) [23].

Finally, patients with lung cancer have a higher risk of recurrent infections, including those opportunistic, due to comorbidities and immunosuppression related to lung cancer therapies and chronic steroid use [24]. The infectious causes of disease can be divided into three categories: bacteria (most commonly Pseudomonas aeruginosa, Stenotrophomonas maltophilia, and Nocardia species), viruses (such as respiratory syncytial virus, parainfluenza virus, influenza virus A and B, and cytomegalovirus), and fungi (including Aspergillus, Fusarium, and Mucorales species, and Pneumocystis jirovecii) [25,26,27].

## 4. Treatment Approaches in Lung Cancer Therapy

The treatment of lung cancer is a rapidly evolving and complex field, with treatment approaches varying based on the type and stage of the tumor, as well as the patient’s overall health. The primary goal is to reduce or eliminate the tumor, manage symptoms, and improve the patient’s quality of life. 

For NSCLC, radical surgical intervention offers the best survival and cure in the early stage [28]. It is less commonly used in SCLC since it is typically diagnosed in the advanced stage [29].

Chemotherapy for lung cancer uses drugs to destroy or slow the growth of cancer cells. This systemic approach is generally conceived to treat all stages of SCLC and is widely employed in NSCLC with an indication in the advanced stage, but more recently, it has been introduced in perioperative settings other than the consolidated indication in adjuvant settings [28,29]. The backbone drugs employed in current clinical practice are platinum or derivative agents. Generally, the preferred strategy is to combine platinum with a second drug, such as tyrosine kinase inhibitors (TKIs) or ICIs, due to their synergistic activities [28,29]. 

Recently, many TKIs acting on specific, actionable genomic alterations (AGAs) in lung cancers have been employed [30]. Generally, these drugs are administered orally and are more effective and less toxic than traditional chemotherapy in selected patients.

ICIs are a new backbone for the treatment of different stages of lung cancer [31]. The proof of principle of these agents is to stimulate the body’s immune system to recognize and attack cancer cells [31]. The use of ICIs, such as PD-1 or PD-L1 inhibitors, has radically transformed the treatment of advanced lung cancer, leading to impressive longer disease control in well-defined subpopulations like the so-called PD-L1 strong positive tumors (tumor proportion score TPS > 50%) [31]. 

Finally, a new class of drugs, the ADCs, are actively studied in all comers immunotherapy refractory/pretreated NSCLC, and now a huge number of clinical trials try to demonstrate their clinical usefulness in the first-line setting in combination with platinum agents and/or ICI [32,33]. 

Instead, radiotherapy uses high-energy radiation to kill cancer cells. It is often combined with surgery or chemotherapy. Radiation may be applied using stereotactic or conventional techniques. Radiofrequency ablation (RFA) and cryoablation are alternatives to radiation [28,29].

In conclusion, lung cancer treatment is personalized based on cancer type, disease stage, and molecular characteristics. Recent advancements in targeted therapies and immunotherapy, among other compounds, have resulted in more effective, less invasive treatments with improved outcomes for many patients, even if lung surgery plus a multimodality approach applied to early/locally advanced stages of the disease remains the best way to definitively cure this cancer.

## 5. Material and Methods

A search of relevant medical literature in the English language, including observational, interventional, and review studies, through September 2024, was conducted in Medline/PubMed and EMBASE databases. Table 2 reports the keywords used to perform the research. Editorials, narratives, conference abstracts, and pre-print publications were excluded. All studies involving ILD patients with lung cancer who experienced AE were included. Three authors (UZ, MF, and VB) searched and screened relevant abstracts and articles independently. When there was a discrepancy between the authors, the articles were collectively discussed, analyzing relevance, strengths, and limitations. 

## 6. AE-ILD Triggered by Bronchoscopic Procedures in Patients with Lung Cancer

Bronchoscopy and different techniques, such as transbronchial cryobiopsy (TBCB) and BAL, are the cornerstone to obtaining tissue samples for histological analysis in lung cancer and ILD diagnosis [34]. Generally, bronchoscopy is a safe procedure, with rare complications such as bleeding and pneumothorax that occur in less than 1% of cases [35]. 

TBCB is a recent technique for diagnosing ILD and a promising procedure for peripheral lung tumors [36]. No data are available regarding the incidence of AE-ILD in patients with lung cancer, even if various studies suggest TBCB offers better diagnostic yield and sample quality than conventional forceps biopsy for lung cancer diagnosis [37].

In 2014, Casoni et al. reported the first case report of AE-ILD after a cryoprobe lung biopsy [38]. The authors recognized that only one patient out of sixty-nine developed AE-ILD after the cryobiopsy. In a larger prospective cohort, Kronborg-White et al. analyzed two hundred and fifty ILD patients who underwent TBCB in a prospective cohort, and only one patient experienced an AE-ILD [39]. In a study involving 106 patients, Bango-Álvarez and colleagues reported no cases of AE-ILD [40]. Similarly, Dhooria et al. found that only three out of one hundred and twenty-eight patients developed AE-ILD after cryoprobe lung biopsy in India [41]. Although the sample size was small, these studies highlighted the limited risk of AE in ILD patients who underwent TBCB.

BAL is also a diagnostic procedure used in the management of lung cancer and ILD [41]. In particular, BAL also plays an important role in diagnosing and managing some ILDs [42]. When combined with clinical data and HRCT imaging, in some cases, BAL can provide enough diagnostic information to avoid invasive procedures [43]. The literature reported different studies about patients with ILD who developed AE after the BAL procedure [44,45,46,47,48]. In a recent retrospective study, Sakamoto et al. analyzed one hundred and twelve IPF patients, of whom four were complicated by AE, of which three cases were fatal [45]. Additionally, the authors conducted a literature review, examining all the characteristics of ILD patients who developed AE after BAL. The patients analyzed were older than 65 (mean age of 66), with a clear predominance of males. Radiologically, most of them exhibited a pattern of usual interstitial pneumonia (UIP). 

In conclusion, bronchoscopic procedures, such as TBCB and BAL, play a central role in diagnosing and treating both cancer and ILD. While generally, they are safe in the general population with less than 1% of complications; some studies show how these procedures could develop AE in up to 3% of cases in patients with ILD. Further studies are needed to assess the impact of bronchoscopy on lung cancer patients with ILD.

## 7. Surgical Lung Biopsies as Triggers for AE-ILD in Lung Cancer Patients

Surgical lung biopsy (SLB) is commonly used to diagnose and stage lung tumors in ILD patients and, at times, to diagnose ILD itself. While complications like pneumothorax and hemorrhage are well-known, the specific risks for ILD patients with lung cancer remain less understood, though they can greatly affect survival [49,50].

In-hospital mortality following SLB can reach 6.4%, with rates ranging between 1.7% for elective and 16% for non-elective cases [51]. Mortality is notably higher in patients with severe or rapidly progressive disease, significant comorbidities, or malignancy [51]. This increased risk is often linked to post-surgery AE in patients with ILD [52]. For instance, Utz et al. found that among 60 ILD patients who underwent SLB, seven died within 30 days due to a following AE [53]. 

Data on AE incidence in ILD patients with concurrent lung cancer undergoing SLB are lacking. However, studies on ILD patients undergoing SLB showed a broad range of outcomes, possibly also in relation to the inclusion of patients with different degrees of severity. Fibla et al. reported a 10.6% 90-day mortality rate in a study of 311 ILD patients, with 23 experiencing AE [54]. In contrast, Kondoh et al. found only a 2% AE incidence in a study of 236 ILD patients, though the associated mortality was high (60%) despite treatment [55].

In conclusion, surgical lung biopsies in ILD patients, especially those with lung cancer, pose significant risks and high postoperative mortality, with the incidence of adverse events still poorly understood.

## 8. Surgical Lung Resection as Triggers for AE-ILD in Lung Cancer Patients

The data on lung resection for lung cancer in ILD patients are more consistent because lobectomy with mediastinal lymph node dissection remains the “gold standard” for treating early-stage NSCLC [56]. While surgery could be curative in patients with lung cancer, the procedure is correlated with an increased risk of postoperative complications and worse survival in ILD patients, also in relation to the occurrence of AE-ILD [7]. 

Sato et al. reviewed data from 108 lung cancer patients with IPF who underwent pulmonary resection, observing that 23% of these patients developed AE-IPF postoperatively, resulting in a 3-year overall survival rate of 34%, with most of the deaths attributed to cancer and AE. The authors emphasized the high incidence of AE and the poor outcomes, suggesting that intensive long-term surveillance is necessary to justify the risks of surgery in these patients [57]. Similarly, another study analyzed 1763 lung cancer patients with ILD who underwent pulmonary resection, showing that 9.3% experienced AE within 30 days post-surgery, with a mortality rate of 43.9%, making AE the leading cause of 30-day mortality [58].

Several risk factors for postoperative complications in ILD patients have been identified, with poor lung function, indicated by lower forced vital capacity (FVC) and/or diffusing capacity of the lungs for carbon monoxide (DLCO), being linked to more severe complications and reduced survival. Furthermore, the presence of a usual interstitial pneumonia (UIP) pattern on a computed tomography (CT) scan is associated with a higher incidence of AE-ILD following surgery [59,60,61]. These findings are supported by Kawasaki et al., who reported increased postoperative morbidity and mortality in IPF patients compared to those with other ILDs. In their study of 711 patients, IPF patients experienced significantly higher rates of pulmonary complications (26% vs. 9.1%) and mortality (8% vs. 0.8%). Additionally, the 5-year survival rate was notably lower for IPF patients (43% vs. 64%) [62].

Surgical techniques and the duration of the intervention can impact AE risk [63]. Surgical interventions lasting over 4 h and extensive lung resections increase the risk [8]. In a study of 101 patients with IPF and lung cancer, 10% experienced AE following lobectomy or sublobar resection [64]. Another study reported a 20% AE rate following pneumonectomy and lobectomy, but no AE occurred after sublobar resection [65]. These findings are supported by a recent meta-analysis, showing that the incidence of postoperative AE in IPF patients with lung cancer was 14%. Notably, sublobar resections were linked to a reduced risk of AE, though the extent of surgical resection did not significantly impact long-term survival. Authors suggested that perioperative strategies, such as screening high-risk cases and opting for sublobar resections, help balance the risk of local recurrence with in-hospital mortality [66]. In this context, minimally invasive techniques such as video-assisted thoracoscopic surgery (VATS) have shown promise in reducing surgical complications in ILD patients [67]. Recent studies suggest that less extensive procedures might provide similar survival outcomes for ILD patients with early-stage lung cancer [68]. However, despite these advancements, ILD patients still experience significantly worse long-term survival rates after lung resection compared to those with lung cancer but without ILD [8].

In summary, while lung resection is often considered a primary option in lung cancer treatment, it may involve the risk of AE in ILD patients, with an incidence reported between 9 and 23%. It is advisable to take a cautious approach and employ strategies such as intensive monitoring and less invasive techniques to reduce these risks.

## 9. Drug-Induced AE-ILD in Lung Cancer Treatment

Chemotherapy is a key component of lung cancer treatment, particularly for those who are not eligible for surgery or have metastatic tumors [69]. However, the choice of adequate systemic therapy is challenging in ILD patients with lung cancer due to the risk of developing AE-ILD [70]. In a recent meta-analysis on ILD patients with NSCLC treated with first-line chemotherapy, Wang et al. reported 8% of AE-ILD, ranging to 5–12% according to the chemotherapy regimen [71].

Before ICIs were available, the standard first-line chemotherapy for lung cancer was usually a platinum-based regimen combined with a histology-specific partner such as paclitaxel, docetaxel, gemcitabine, or etoposide [72]. In Japan, a nationwide surveillance study assessed the risk of AEs following chemotherapy in patients with ILD [73]. This study included 396 lung cancer patients who received first-line chemotherapy between 1990 and 2009. Among these patients, 13% experienced AEs [73]. The most common first-line treatments were carboplatin plus paclitaxel and carboplatin plus etoposide, with AE incidence rates of 8.6% and 3.7%, respectively [73]. A subsequent retrospective study of 278 patients who received second-line chemotherapy between 2002 and 2012 found an AE incidence rate of 16.2% [73]. Two Japanese clinical trials have investigated the efficacy and safety of carboplatin combined with nab-paclitaxel in ILD patients with NSCLC. One study by Kenmotsu et al., which analyzed 94 patients with lung cancer and ILD, found that only 4% developed AE-ILD after therapy [74]. Another similar study involving thirty-six patients reported only two cases of AE-ILD after chemotherapy, with one resulting in death [75]. The incidence of AE-ILD is higher in patients treated with second-line agents such as docetaxel and gemcitabine, with rates of 28% and 43%, respectively [76]. 

Targeted therapies, such as EGFR and ALK inhibitors, are increasingly being used to treat NSCLC [77]. However, these therapies come with an increased risk of pneumonitis in patients with ILD [78]. For instance, a study of over 3000 Japanese NSCLC patients revealed that 4% developed AE-ILD within 12 weeks of gefitinib treatment [79]. Similarly, a meta-analysis of over 2000 NSCLC patients treated with ALK inhibitors found a 2% incidence of AE [80].

The introduction of ICIs has significantly improved overall survival in lung cancer patients, though immune-related side effects remain a concern. While the risk of pneumonitis is well-documented, the incidence of AE in ILD patients is still unclear due to the limited number of small retrospective studies [81]. For example, Nishiyama et al. found that 14.5% of 48 NSCLC and ILD patients treated with nivolumab, pembrolizumab, or atezolizumab developed AE-ILD [82]. In another recent study by Takahara et al., almost 50% of 27 patients with ILD and lung cancer experienced AE [83].

Few studies evaluated AE-ILD incidence in patients with lung cancer treated with a combination of CT and ICIs. A recent multicenter prospective study enrolled 21 patients with extensive-stage SCLC and ILD treated with a combination of carboplatin-etoposide and durvalumab. The authors showed that AE-ILD incidence during induction treatment was only 9.5%, similar to that of patients treated with chemotherapy alone [84]. 

Regarding ADCs, some side effects are reported for trastuzumab ILD patients, but data are limited, and ADCs are actually contraindicated in all ILD patients with NSCLC [85]. 

Recent studies underlined the promising role of biomarkers in identifying cancer patients with ILD who are at higher risk of AEs during systemic therapy [86,87,88]. Krebs von den Lungen-6 (KL-6) has been associated with an increased risk of AE in ILD patients with lung cancer [87,88]. Further research is essential to standardize these biomarkers and establish risk thresholds to improve monitoring and facilitate early intervention.

In conclusion, while chemotherapy is important in treating lung cancer, its use in patients with concomitant ILD carries significant risks. Looking for the best risk/benefit ratio and balancing treatment efficacy with the potential for severe adverse events, especially AE-ILD, is a challenge, and most importantly, the multidisciplinary team is essential to deciding the best way to treat this special population.

## 10. Radiotherapy and the Risk of AE-ILD in Lung Cancer Patients

Radiotherapy (RT) plays a central role in the treatment of lung cancer. Lung cancer is the second most common indication for RT globally and has consistently been demonstrated to be the most common indication for palliative RT [89,90,91]. Chemo-radiotherapy, followed by maintenance immunotherapy, is the standard of care for the treatment of advanced NSCLC. While radiotherapy is generally well tolerated, the side effects can vary depending on the total dose, the treatment area, and size [92]. Even if most studies have focused more on radiation pneumonitis, one of the side effects in ILD patients is the occurrence of AE [93]. 

The incidence of AE-ILD in patients with lung cancer treated with chemo-radiotherapy varies widely, ranging from 6% to 83% [94]. In a retrospective study with one hundred and twenty-two patients enrolled, Koyama et al. reported that seven patients with SCLC were treated with chemo-radiotherapy [95]. Data showed that five of them reported an AE-ILD. On the other hand, Taya et al. showed a lower incidence, with only one out of fifteen patients experiencing AE-ILD during chemo-radiotherapy [96].

Kim et al. studied 101 lung cancer patients with IPF and found that 17.9% who received radiation therapy experienced AE [64]. Patients with AE had a median survival of five months, compared to sixteen months for those without AEs [64].

Chen et al. conducted a systematic review of the incidence of AE-ILD in treatments for early-stage non-small cell lung cancer (ES-NSCLC) with coexisting ILD [97]. They found that stereotactic ablative radiation therapy (SABR) had high rates of treatment-related mortality and toxicity, especially in patients with IPF (15% vs. 33% and 25% vs. 71%) [97].

In contrast, particle beam therapies, including proton beam therapy (PBT) and carbon ion beam therapy (CIBT), demonstrated a lower incidence of treatment-related mortality (4.3%) and toxicity (18.2%) compared to SABR [98]. A retrospective study by Hyun et al. investigated the relationship between AEs and treatment modalities in lung cancer patients with ILD. They discovered that 16.2% of those who received radiotherapy experienced adverse events, which were linked to poorer survival outcomes compared to patients without adverse events [86].

In conclusion, RT in lung cancer patients with ILD faces a higher risk of AE. Newer treatments like PBT and CIBT show promising results in reducing mortality and toxicity compared to chemo-radiotherapy and SABR.

## 11. AE-ILD Risk Following Percutaneous Ablation of Lung Cancer Tumors

Image-guided tumor Ablation (IGTA), including RFA and cryoablation, has emerged as an important treatment option for patients with lung cancer who are not suitable candidates for surgery due to advanced disease, age, or other comorbidities [99]. IGTA utilizes thermal or electrical methods to destroy tumor cells directly and is primarily performed under CT guidance [100]. These minimally invasive procedures offer palliative benefits by alleviating pulmonary symptoms in patients who have limited treatment options, mainly when systemic chemotherapy is too dangerous or has failed [101]. The experience with the use of IGTA in ILD patients with lung cancer is limited. However, in the general population, the procedure is generally safe. It is associated with pneumothorax, although it is not associated with increased mortality. In an observational study, Dupuy et al. found that the 2-year survival rates in 54 patients were similar to those historically reported for stereotactic body radiotherapy (SBRT) [102]. Kashima and colleagues described the use of IGTA in ILD patients [103]. In their retrospective analysis, three out of forty-two patients with lung cancer and ILD died of AE-ILD [103]. A recent systematic review analyzed 46 patients with ILD who were treated with RFA for lung cancer. The review reported an ILD-specific toxicity rate of 25% and a 9% mortality rate [104]. Finally, Yamauchi et al. reported that 2 out of 11 patients with IPF (18%) experienced AE-ILD and subsequent death after percutaneous cryoablation for lung cancer [105]. In conclusion, IGTA is an emerging treatment supported by promising preliminary data. However, further studies are necessary to validate these results, and the risk of AE needs to be considered in patients with concomitant ILD.

## 12. Treatment of AE-ILD

Since no evidence-based management strategy is available for AE-ILD, as well as for AE-IPF, the currently used approach is based on supportive care and unproven drugs [18]. Thus, the IPF guidelines weakly recommend the use of systemic steroids in patients with AE-IPF, with no recommendation on the most appropriate type, dose, and duration of steroids to be used [106]. However, in a recent retrospective study, no evidence was found that corticosteroid use improves the outcomes of patients with IPF admitted because of an AE [107]. Currently, two prospective studies are ongoing, the first exploring the efficacy of glucocorticoids versus placebo for the treatment of AE-IPF (EXAFIP2) [108], the other evaluating the efficacy of pulse steroid for 3 days with methylprednisolone 10 mg/kg daily followed by background steroid therapy compared to background steroid therapy [109].

In 2016, Collard and colleagues recommended that immunosuppressive therapy should be studied in randomized controlled trials to better evaluate their possible benefit in AE-IPF [18]. In a recent randomized, placebo-controlled trial, Naccache and colleagues showed that intravenous pulses of cyclophosphamide (CYC) added to high-dose glucocorticoids did not reduce all-cause mortality in patients with AE-IPF. In contrast, a trend towards increased mortality at 3 months, although less pronounced after 6 months, was observed in patients receiving CYC compared with placebo [110].

Nevertheless, in a pilot study, patients affected by AE-IPF were treated with therapeutic plasma exchange and rituximab (RTX), a chimeric mouse-human anti-CD20 antibody, supplemented in some cases with intravenous immunoglobulins (IVIGs) [111].

Seven patients treated with RTX combined with therapeutic plasma exchange demonstrated a one-year survival rate of 46% compared to 5% in historical controls treated only with corticosteroids, particularly when IVIGs were added [111]. These results will be further evaluated in an ongoing, prospective, multicenter, randomized controlled trial [112].

Although excluding an infectious trigger is not required to diagnose AE-ILD, detecting possible pathogens remains important to choosing the most appropriate antibiotic treatment. 

Therefore, considering the difficulties in differentiating AE-ILD, and particularly AE-IPF, from a bronchopulmonary infection, broad-spectrum antibiotics are often administered in patients with acute worsening of dyspnea [113].

Although most patients with AE-IPF develop acute respiratory failure, current treatment guidelines suggest that these patients should not receive mechanical ventilation (MV) based on the futility of the intervention. However, in more recent studies, the overall survival of some patients improved [114], and possible predictive factors for mortality associated with MV have recently been investigated, but further studies are required to correctly select the subgroup of patients to be treated [115].

Nevertheless, considering the very frequent unfavorable outcomes, palliative care appears to be an important treatment for these patients, considering that most of them generally are not intubated. Palliation of dyspnea and anxiety is generally treated with opioids and benzodiazepines [116,117]. Respiratory depression is often wrongly considered a major clinical problem; nevertheless, a discussion with patients and relatives is mandatory to explain the aims of palliation [118]. Cough management, which is also important, can be done by using various types of drugs, including opioids, to palliate this symptom [116].

## 13. Limitation

This narrative review has some limitations that should be considered. First of all, since the prevalence and impact of AE in ILD patients with lung cancer is very limited and heterogeneous, most of the studies used were retrospective and with a small sample size. Secondly, the studies analyzed may refer to a different definition of AE, as the definition of AE was updated recently and included different populations of patients with ILD. Finally, we did not perform a formal assessment of the quality of the studies.

## 14. Conclusions

In conclusion, AE-ILD remains a critical and challenging complication in lung cancer patients from diagnosis to treatment. According to the revised 2016 criteria, AE-ILD is most extensively studied in IPF, but it is also observed in other ILDs, often applying the same criteria due to clinical similarities. As summarized in Table 3, the risk of AE is further influenced by diagnostic and therapeutic interventions, such as bronchoscopy, surgical procedures, chemotherapy, and radiotherapy, all of which can precipitate AE-ILD in susceptible patients. Despite advances in diagnostic techniques and treatment strategies, AE-ILD continues to carry a high mortality risk, underscoring the need for monitoring and a cautious approach to managing ILD patients, mainly when invasive procedures are considered. Future research should focus on understanding the mechanisms behind AE-ILD across various types, not just IPF. This could lead to more tailored treatments. Longitudinal studies are also needed to evaluate alternative treatment strategies that may reduce AE-ILD risk in lung cancer patients undergoing invasive procedures. Moreover, identifying predictive biomarkers for AE-ILD could help clinicians better anticipate and manage risks before invasive treatments. As research progresses, a better understanding of the mechanisms driving AE-ILD and the development of tailored therapeutic approaches are essential to improve outcomes for these patients.

## Figures and Tables

**Table 1:** Main features of differential diagnosis of acute exacerbations of interstitial lung diseases.

	AE–ILD	Drug–Induced Toxicity	Radiation Pneumonia	Lung Infection
**Onset**	Typically less than 1 month	Either within weeks or delayed	3–12 weeks	Acute
**Symptoms**	Dyspnea, cough (with or without sputum production), fever and flu-like symptoms.	Dyspnea, fever, and peripheral eosinophilia. Rare hemoptysis, and anaemia.	Nonproductive cough, dyspnea, and chest pain.	Productive cough, dyspnea and fewer.
**BAL**	Useful for differential diagnoses.	Eosinophilic/lymphocytic.	Lymphocytic.	Neutrophilic.
**CT-pattern**	Bilateral GGos or consolidative opacities over an ILD.	Non specific. Major radiological pattern are: NSIP, OP, DAD, and simple pulmonary eosinophilia. UIP is rare.	GGos and/or airspace consolidation.	Airspace opacities. PJP and CMV can be interstitial opacities.
**Prognosis**	Mortality ranging from 50% to 80%	Early drug discontinuation leads to recovery. Delayed diagnosis risks ARDS.	Improving within 3–18 months	Variable depending on patient and infection.

AE: Acute Exacerbations; ILD: Interstitial Lung Diseases; BAL: Bronchoalveolar Lavage; CT: Computed Tomography; GGos: Ground-Glass Opacities; NSIP: Nonspecific Interstitial Pneumonia; OP: Organizing Pneumonia; DAD: Diffuse Alveolar Damage; UIP: Usual Interstitial Pneumonia; PJP: Pneumocystis Jiroveci Pneumonia; CMV: Cytomegalovirus; ARDS: Acute Respiratory Distress Syndrome.

**Table 2:** Keywords used to perform the research.

- Interstitial Lung Diseases (OR ILD OR Diffuse Parenchymal Lung Diseases OR IPF) AND Acute Exacerbations (OR AE-ILD OR AE) AND Bronchoscopy (OR BAL OR Cryoprobe); - Interstitial Lung Diseases (OR ILD OR Diffuse Parenchymal Lung Diseases OR IPF) AND Acute Exacerbations (OR AE-ILD OR AE) AND Surgical Lung Biopsy (OR SBL OR Surgery); - Interstitial Lung Diseases (OR ILD OR Diffuse Parenchymal Lung Diseases OR IPF) AND Acute Exacerbations (OR AE-ILD OR AE) AND Chemotherapy (OR ICIs OR Immune Checkpoint Inhibitors); - Interstitial Lung Diseases (OR ILD OR Diffuse Parenchymal Lung Diseases OR IPF) AND Acute Exacerbations (OR AE-ILD OR AE) AND Radiotherapy (OR Stereotactic Body Radiation Therapy OR Stereotactic Ablative Radiation Therapy OR Proton Beam Therapy OR Carbon Ion Beam Therapy); - Interstitial Lung Diseases (OR ILD OR Diffuse Parenchymal Lung Diseases OR IPF) AND Acute Exacerbations (OR AE-ILD OR AE) AND Percutaneous Ablation (OR IGTA OR RFA OR MWA);

ILD: Interstitial Lung Diseases; IPF: Idiopathic Pulmonary Fibrosis; AE: Acute Exacerbations; BAL: Bronchoalveolar Lavage; SBL: Surgical Lung Biopsy; ICIs: Immune Checkpoint Inhibitors; IGTA: Image-Guided Tumor Ablation; RFA: Radiofrequency Ablation; MWA: Microwave Ablation.

**Table 3:** Summary of incidence of AE-ILD and AE-IPF associated with medical procedures and oncological treatments.

Procedures	Incidence of AE-ILD	Incidence of AE-IPF	References
**Bronchoscopic procedures**			
**TBCB**	0–2%	-	Casoni et al. [38], Kronborg-White et al. [39], Bango-Alvarez et al. [40], Dhooria et al. [41]
**BAL**	-	3%	Sakamoto et al. [45]
**Surgical Procedures**			
**SLB**	2–7%	11%	Utz et al. [53], Fibla et al. [54], Kondoh et al. [55]
**SLR**	9%	23%	Sato et al. [58], Sato et al. [57]
**Systematic Therapy**			
**Chemotherapy**	5–12%	-	Wang et al. [71]
**TKIs**	4%	-	Kudoh et al. [79]
**ALK inhibitors**	2%	-	Suh et al. [80]
**ICIs**	14–50%	-	Nishiyama et al. [82], Takahara et al. [83]
**Radiotherapy**			
**SBRT**	-	17%	Kim et al. [64]
**SABR**	33%	71%	Chen et al. [97]
**Particle beam therapy**	18%	-	Vyfhuis et al. [98], Hyun et al. [86]
**Percutaneous Ablation**			
**IGTA**	7%	-	Kashima et al. [103]
**RFA**	25%	-	Chen et al. [104]
**Cryoablation**	18%	-	Yamauchi et al. [105]

AE: Acute Exacerbations; ILD: Interstitial Lung Diseases; IPF: Idiopathic Pulmonary Fibrosis; TBCB: Transbronchial Cryobiopsy; BAL: Bronchoalveolar Lavage; SLB: Surgical Lung Biopsy; SLR: Surgical Lung Resection; TKIs: Tyrosine Kinase Inhibitors; ALK: Anaplastic Lymphoma Kinase; ICIs: Immune Checkpoint Inhibitors; SBRT: Stereotactic Body Radiotherapy; SART: Stereotactic Ablative Radiation Therapy; IGTA: Image-Guided Tumor Ablation; RFA: Radiofrequency Ablation.

## Data Availability

All data generated or analyzed during this study are included in this article. Further inquiries can be directed to the corresponding author.
